# Age-Dependence of Femoral Strength in White Women and Men

**DOI:** 10.1359/jbmr.091033

**Published:** 2009-10-26

**Authors:** Tony M Keaveny, David L Kopperdahl, L Joseph Melton, Paul F Hoffmann, Shreyasee Amin, B Lawrence Riggs, Sundeep Khosla

**Affiliations:** 1 Departments of Mechanical Engineering and Bioengineering, University of California–Berkeley Berkeley, CA, USA; 2 O. N. Diagnostics Berkeley, CA, USA; 3 Division of Epidemiology, Department of Health Sciences Research, College of Medicine, Mayo Clinic Rochester, MN, USA; 4 Division of Rheumatology, Department of Internal Medicine, College of Medicine, Mayo Clinic Rochester, MN, USA; 5 Division of Endocrinology, Department of Internal Medicine, College of Medicine, Mayo Clinic Rochester, MN, USA

**Keywords:** bone strength, osteoporosis, aging, finite-element analysis, biomechanics

## Abstract

Although age-related variations in areal bone mineral density (aBMD) and the prevalence of osteoporosis have been well characterized, there is a paucity of data on femoral strength in the population. Addressing this issue, we used finite-element analysis of quantitative computed tomographic scans to assess femoral strength in an age-stratified cohort of 362 women and 317 men, aged 21 to 89 years, randomly sampled from the population of Rochester, MN, and compared femoral strength with femoral neck aBMD. Percent reductions over adulthood were much greater for femoral strength (55% in women, 39% in men) than for femoral neck aBMD (26% in women, 21% in men), an effect that was accentuated in women. Notable declines in strength started in the mid-40s for women and one decade later for men. At advanced age, most of the strength deficit for women compared with men was a result of this decade-earlier onset of strength loss for women, this factor being more important than sex-related differences in peak bone strength and annual rates of bone loss. For both sexes, the prevalence of “low femoral strength” (<3000 N) was much higher than the prevalence of osteoporosis (femoral neck aBMD *T*-score of −2.5 or less). We conclude that age-related declines in femoral strength are much greater than suggested by age-related declines in femoral neck aBMD. Further, far more of the elderly may be at high risk of hip fracture because of low femoral strength than previously assumed based on the traditional classification of osteoporosis. © 2010 American Society for Bone and Mineral Research.

## Introduction

Osteoporosis is an underdiagnosed and undertreated disease, hip fracture being its most severe sequela.(1) Low areal bone mineral density (aBMD) at the femoral neck, as measured by dual-energy X-ray absorptiometry (DXA), is a powerful determinant of hip fracture(2) and the recommended tool for risk assessment.(3) However, most osteoporotic fractures occur in individuals who do not have osteoporosis by DXA criteria.(4–7) While a number of non-BMD-related factors may account for this, aBMD is inherently limited in assessing femoral strength because of its 2D nature and inability to quantify specific bone compartments or structures.(8) A more refined clinical characterization of femoral strength and its dependence on age therefore may improve our understanding of hip fracture etiology and lead to better strategies for reducing the burden of this important clinical problem.(9)

Finite-element analysis of quantitative computed tomographic (QCT) scans—termed here *biomechanical CT* (BCT)—is the most technologically advanced method currently available for noninvasive clinical assessment of femoral strength. This technique has been well validated in cadaver studies(10–12) and has been used to provide unique insight into osteoporosis therapies.(13–16) In a recent prospective fracture surveillance study of elderly men,(17) all men with a BCT-derived femoral strength value below 2900 N suffered a new hip fracture, suggesting more generally that those with “low femoral strength” (<3000 N) are at high risk of fracture. Further, over half those who fractured with such low femoral strength were classified as having osteopenia rather than osteoporosis on the basis of their DXA-derived aBMD *T*-score.

The goal of this cross-sectional study was to apply BCT to a population-based cohort in order to (1) characterize the variations in femoral strength across age among adult women and men, (2) estimate the prevalence of low femoral strength (<3000 N), and (3) compare these trends with those for femoral neck aBMD and the prevalence of osteoporosis (femoral neck aBMD *T*-score of −2.5 or less).

## Methods

### Subjects

We analyzed CT data obtained previously from an age-stratified random sample of Rochester, MN, residents.(18) This community is highly characteristic of the US white population, but blacks and Asians are underrepresented.(19) The sample spanned ages from 20 to 97 years and included 375 women and 325 men. Reflecting the ethnic composition of the community, 98% of the subjects were white. Thirty-two percent of the men and 29% of the women were obese, as defined by a weight greater than 30% of ideal for their height. Ninety-four postmenopausal women were receiving estrogen therapy, and 6 postmenopausal women and 3 men were receiving bisphosphonate therapy for osteopenia. Because of the large number of postmenopausal women receiving estrogen at the time of recruitment, we oversampled in the 50- to 69-year age range to have adequate numbers of untreated women for analysis. There was an offsetting undersampling of young adult women and men.

As described in detail elsewhere,(18) single-energy QCT scans were made at the proximal femur using a multidetector CT scanner (Light Speed QX-I, GE Medical Systems, Wakesha, WI, USA) with a slice width of 2.5 mm and an in-plane voxel size of 0.74 mm. After deleting scans (*n* = 17) that had image artifacts that prevented us from performing accurate BCT analysis, 362 women (ages 21 to 97 years) and 317 men (ages 22 to 93 years) remained in the study.

### Femoral strength and aBMD measurements

**Fig. 1:**
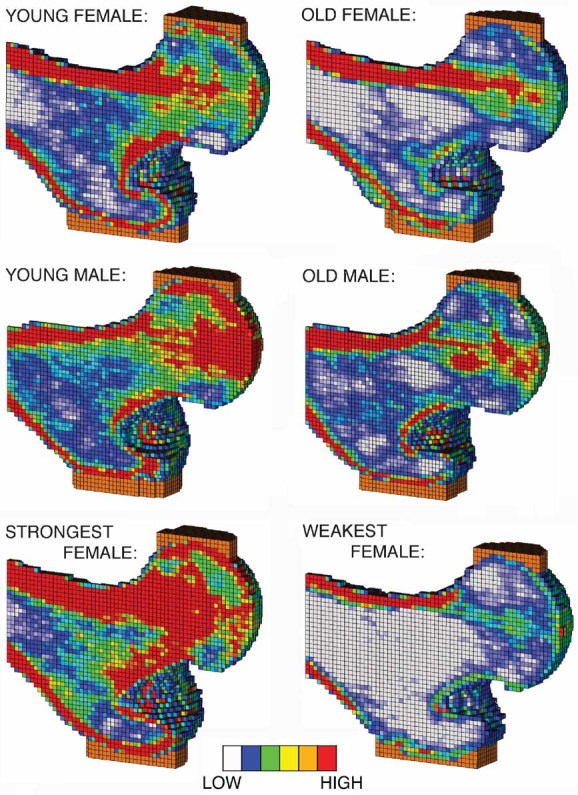
Finite-element models for six subjects: a typical young and old woman and man, as well as the strongest and weakest women in the cohort. The images show local regions of high (*red*) and low (*blue*) strength bone. The bone is virtually loaded in a typical sideways fall configuration through the virtual PMMA plates (colored orange) shown at the head and greater trochanter.

The two main outcomes of our analysis were the BCT-derived estimate of femoral strength (N) and the quantitative CT-derived measure of femoral neck aBMD (g/cm^2^). To estimate femoral strength, as described in detail elsewhere,(15–17) each QCT scan was converted into a 3D finite-element model of the proximal femur (Fig. 1) in which the local material properties of cortical and cancellous bone were assigned from the spatially varying calibrated Hounsfield units from the CT scan using empirically derived relations.(20–22) Each patient-specific finite-element model then was virtually loaded to failure in simulation of an unprotected sideways fall with impact on the greater trochanter. Nonlinear analyses were used in these simulations, the result being an estimate of the strength of the whole proximal femur. Laboratory experiments on 76 elderly cadavers loaded in a sideways fall configuration at high speed have shown a high correlation (*r*^2^ = 0.78) and *Y* = *X* type agreement between such estimates of femoral strength and direct measures from biomechanical testing.(12) To measure femoral neck aBMD, the QCT scan was projected into the plane of a standard clinical anteroposterior DXA hip exam. A direct comparison of this CT-equivalent measure of aBMD with a DXA-measured value was possible for 100 randomly chosen subjects for whom good-quality CT and DXA scans were both available at a later date, and these data confirmed the validity of the CT-derived measure (*r*^2^ = 0.95). The CT-derived measures were scaled linearly to provide Lunar-equivalent values.

### Statistical analysis

Means and 95% confidence intervals (CIs) for femoral strength and femoral neck aBMD were calculated per decade of age, except that the two youngest and two oldest groups were separately pooled because there were not as many 20 to 29 and 90 + year-olds. Linear regression analysis was used on these mean values to characterize average age-related changes in femoral strength. In these regressions; we fit only the line to age > 45 years for women because there was no significant change before then in the mean values; for men, we fit the line to age > 55 years for the same reason. For comparison purposes, these same age ranges were used to describe the age-related changes in femoral neck aBMD. The resulting linear regression equations relating strength (or density) to age then were used analytically to estimate the average annual percent change in femoral strength and femoral neck aBMD from these cross-sectional data. The percent change at each year was calculated as the change in strength over each single year divided by the value at the start of that year. To characterize the prevalence of low femoral strength, we calculated the proportion of the cohort in each age group having a femoral strength below 3000 N. This cut point was based on our previous analysis—using the same implementation of the BCT analysis technique as used here—of incident hip fracture in elderly men(17); without exception, all men in that prospective study who had a femoral strength of less than 2900 N suffered a new hip fracture. Similarly, the prevalence of femoral neck aBMD *T*-scores of −2.5 or less was estimated in each age group. These *T*-scores were calculated for both sexes using female young reference values(23) for Lunar DXA in the femoral neck region (0.98 ± 0.12 g/cm^2^).(24) Comparisons of femoral neck aBMD with age also were made against equivalent data from Lunar and Hologic reference values in order to confirm that our cohort was representative of the larger US white population. The Hologic data were based on the third National Health and Nutrition Examination Survey (NHANES III).(25) To enable comparison of our results with other prevalence studies, for each outcome the prevalence rate for those aged 50 years and above was age adjusted to the demographic structure of the US white population aged 50 years and over in 2000.

## Results

**Fig. 2:**
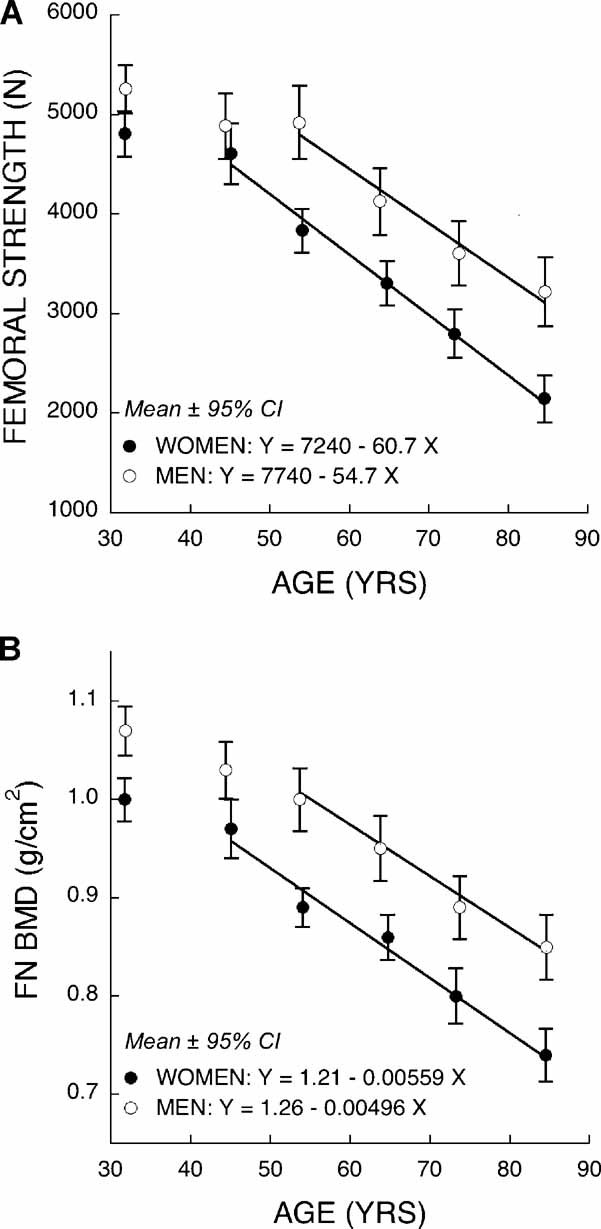
Mean (±95% CI) values of femoral strength (*A*) and femoral neck (FN) aBMD (*B*) by decade of age for Rochester, MN, women and men. Data for subjects in the 20- to 39-year age range and over age 80 were pooled to account for the smaller sample size in those groups (see Table 1 for sample sizes). Linear regression analysis of these mean data over the range of the best-fit lines was used to estimate age dependent rates of loss (for women over age 45 years and men over age 55 years).

**Fig. 3:**
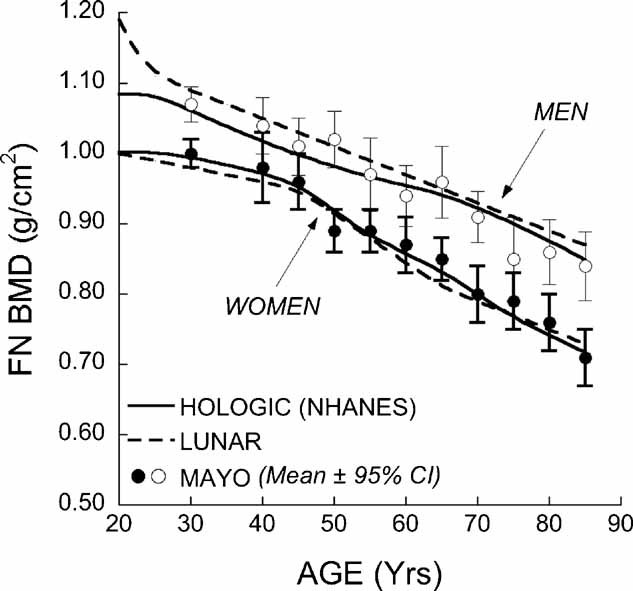
Comparison with published manufacturer/reference values(24) for femoral neck (FN) aBMD and FN aBMD as measured in this study for the Rochester cohort (mean ± 95% CI). The Hologic data, derived from NHANES III,(25) were converted to Lunar-equivalent (L-equiv) values using the following equation: Lunar = 0.142 + 1.013 × Hologic.(24) Trends lines are shown for the Hologic and Lunar data sets. For both sexes, both manufacturer data sets fall within the 95% CI of the Rochester figures.

Femoral strength and femoral neck aBMD varied in similar ways with age for each sex, with notable declines starting in the mid-40s for women and a decade later for men (Fig. 2). Once these declines started, the linear regression analysis of these cross-sectional data indicated that there were slightly higher (∼10%) estimated rates of annual (absolute) loss in femoral strength in women compared with men (61 versus 55 N/year). Similar trends were observed for femoral neck aBMD, and as expected, the estimated average rate of loss was slightly higher (∼7%) in women than in men (5.6 versus 5.2 g/cm^2^/year). Comparison of the means and standard deviations of the femoral neck aBMD values by each decade of age for our Rochester cohort against the published reference values from the Lunar and Hologic DXA manufacturers confirmed the representativeness of our cohort (Fig. 3).

**Fig. 4:**
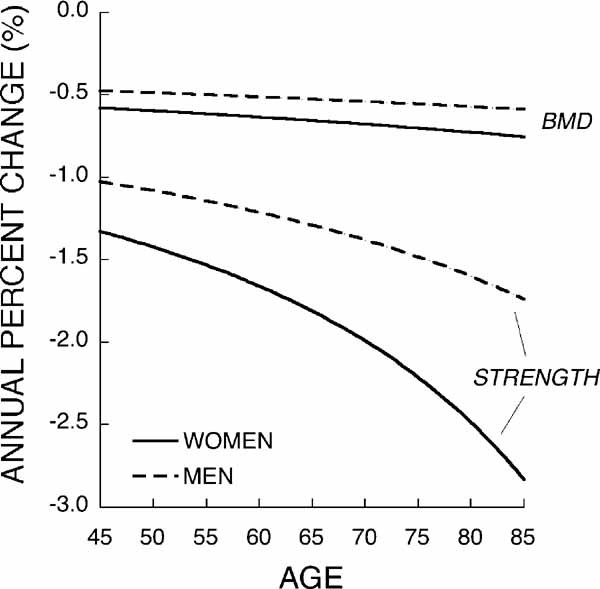
Estimated annualized percent change in femoral strength and femoral neck aBMD for Rochester, MN, women and men, as calculated from linear regression analysis of the data shown in Fig. 2.

Despite these apparently similar age trends for femoral strength and femoral neck aBMD, annual percent reductions in the most elderly women were over threefold greater for femoral strength than for femoral aBMD (Fig. 4). The percent reductions in femoral strength for the oldest group (mean age approximately 85 years) with respect to the youngest group (mean age approximately 30 years) were 55% and 39% for women and men, respectively—about twice the size of the reductions in femoral neck aBMD (26% and 21%, respectively). Using the linear regression equations shown in Fig. 2 to calculate annual percent changes, starting from the first decade at which reductions occurred, the estimated annual percent reduction in femoral strength for women ranged from 1.3% at age 45 to 2.8% at age 85; for femoral neck aBMD, these respective values were only 0.6% (age 45) and 0.8% (age 85). For men, femoral strength did not decrease until one decade later; the estimated annual percent reductions at ages 55 and 85 years were 1.1% and 1.7%, respectively, for femoral strength, over twice those for femoral neck aBMD (0.5% and 0.6%).

**Fig. 5:**
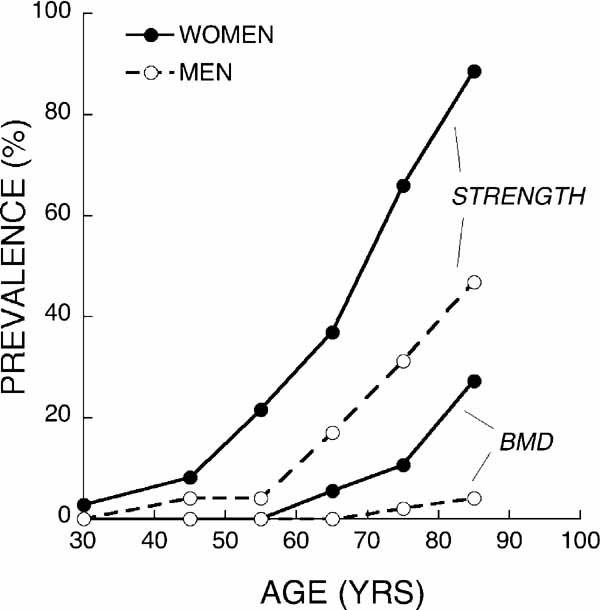
Age-specific prevalence of “low femoral strength” (femoral strength < 3000 N) and osteoporosis (femoral neck aBMD *T*-score ≤ –2.5) for Rochester, MN, women and men.

For both sexes for each age, the prevalence of low femoral strength (<3000 N) was much higher than the prevalence of osteoporosis (*T*-score ≤ −2.5; Fig. 5). The overall age-adjusted prevalence for women 50 years of age and older was 43.9% for low femoral strength compared with 7.2% for osteoporosis; for men, these values were 18.9% and 1.0%, respectively. The prevalence of low femoral strength became appreciable (>15% to 20%) at the fifth decade for women and a decade later for men and then increased with age at similar gradients in both sexes, at least initially. However, unlike the rather uniform age trends for mean values of femoral strength, there was an additional increase in the prevalence of low femoral strength for women compared with men starting in the seventh decade. By the eighth decade, the prevalence of low femoral strength, almost twofold higher for women (89%) than for men (47%), was substantially greater than the prevalence of osteoporosis (27% for women, 4% for men).

The prevalence of osteoporosis was similar regardless of whether the young reference value from the Lunar and the Hologic manufacturer was used. It was higher when the young reference value for the Rochester cohort itself was used but still lower than the prevalence of low femoral strength (Table 1). The prevalence of low femoral strength remained higher than the prevalence of osteoporosis (based on the manufacturers' reference values) even if it was defined as a femoral strength value less than 2000 N.

**Table 1:** Prevalence (in Percent) of Osteoporosis (Femoral Neck aBMD *T*-Score ≤ –2.5) Using Different (Female) Young Reference Values for the *T*-Score Calculation (Lunar or Hologic Published Reference Values, Rochester Cohort-Specific Reference Value) and Prevalence of Low Femoral Strength (Defined by Strength Values less than Either 2000 or 3000 N) Among Rochester, MN, Women and Men

		Osteoporosis (%)			Low femoral strength (%)	
			
Age group	Number of subjects	Lunar^a^	Hologic^a^	Rochester^a^	<2000	<3000
*Women*						
20–39	75	0.0	0.0	1.3	1.3	2.7
40–49	49	0.0	0.0	2.0	0.0	8.2
50–59	74	0.0	0.0	6.8	2.7	21.6
60–69	73	5.5	6.8	16.4	8.2	37.0
70–79	47	10.6	12.8	38.3	17.0	66.0
>80	44	27.3	31.8	70.5	50.0	88.6
Age-adjusted^b^	238	7.2	8.6	24.3	13.3	43.9
*Men*						
20–39	75	0.0	0.0	0.0	0.0	0.0
40–49	49	0.0	0.0	0.0	0.0	4.1
50–59	49	0.0	0.0	0.0	0.0	4.1
60–69	47	0.0	0.0	2.1	0.0	17.0
70–79	48	2.1	6.3	20.8	8.3	31.3
>80	49	4.1	6.1	32.7	16.3	46.9
Age-adjusted^b^	193	1.0	2.1	9.3	3.9	18.9

a Young reference values (mean ± SD) were 0.98 ± 0.12 and 0.85 ± 0.11 g/cm^2^ for Lunar and Hologic, respectively, and 1.00 ± 0.09 g/cm^2^ (in Lunar-equivalent values) for the Rochester cohort. A mean ± SD value of 0.85 ± 0.11 g/cm^2^ for femoral neck aBMD on a Hologic densitometer is equivalent to 1.00 ± 0.11 g/cm^2^ on a Lunar device.

b For those aged 50 years and older, values were age adjusted to the total population distribution of US whites aged over 50 years in 2000.

## Discussion

Femoral neck aBMD is correlated with bone strength(26) and is the preferred clinical metric for assessing both fracture risk(3) and the prevalence of osteoporosis.(23) However, our results demonstrate that femoral strength is reduced to a much greater extent during adulthood than would be suggested by reductions in femoral neck bone density and that this effect is accentuated in elderly women. We also found that the prevalence of low femoral strength, as defined in this study (<3000 N), was much greater in this cohort than was the prevalence of osteoporosis. Our prevalence threshold for femoral strength was based on our observation that all men in the MrOS prospective fracture surveillance study who had BCT-derived femoral strength values of less than 2900 N reported a new hip fracture during follow-up.(17) Additionally, the hazard ratio for hip fracture per standard deviation decrease in femoral strength in that study was large (13.1, 95% CI 3.9–43.5). The MrOS study did not include women, and no similar studies for women have yet been reported. Nonetheless, assuming that a femoral strength below 3000 N places any individual at high risk of a hip fracture, our results suggest that far more of the elderly may be at high risk of hip fracture because of low femoral strength than previously assumed based on the traditional femoral neck *T*-score-based classification of osteoporosis.(23) While the incidence of hip fractures in the elderly at advanced age is much less than the prevalence of reduced femoral neck strength reported here, this is so because hip fractures rarely occur without a fall(27,28); indeed, a fall generally can be considered as a necessary condition for a hip fracture.(14) Despite the important etiologic role of falls for hip fracture in the elderly, this increased prevalence of those with low femoral strength compared with those with low femoral neck aBMD may partially explain why known reductions in femoral neck aBMD predict only a doubling of hip fracture risk between the ages of 60 and 80 years instead of the 13-fold increase actually observed.(29)

The age-dependent reduction in aBMD as reported from NHANES III(25) supports the generality of our study data. In NHANES III, average femoral neck aBMD in white women decreased from 0.86 g/cm^2^ at age 25 years to 0.57 g/cm^2^ at age 80 years. This rate of decrease (∼0.55% per year) is entirely consistent with our results. However, overall prevalence values depend on the cutoff value used to define osteoporosis,(30,31) and as shown in Table 1, our results were sensitive to this parameter. This sensitivity was mainly due to the slightly smaller standard deviation in aBMD for the Rochester young reference group than for the manufacturer cohorts (Table 1), presumably owing to the more uniform nature of the Rochester cohort compared with that used in NHANES III. It also may have been influenced by having a single experienced technician make all the CT measurements in the Rochester cohort. Moreover, such CT measurements enable more standardized positioning of the femoral neck during calculation of aBMD values and thus introduce fewer random measurement errors from misalignment. We chose to follow clinical guidelines and thus defined *T*-scores using a manufacturer-specified value for a young female reference for both sexes.(23)

Likewise, the cut point chosen to define “low femoral strength” was based on our observations among elderly men in MrOS, in which all men with a femoral strength of less than 2900 N fractured their hip during the mean 5.6-year surveillance period.(17) It is possible that factors associated with the CT scanning protocol may alter this value in other study populations, or the value may be different in women. However, BCT analysis in two drug studies on osteoporotic postmenopausal women both reported average values of femoral strength at baseline of about 2500 N.(15,16) These two studies used the same techniques as employed here for the finite-element modeling but employed different instruments and scan-acquisition protocols for the CT scanning. Since the entry criteria in these drug studies were designed to include only women at high risk of fracture using well-accepted clinical criteria, these data suggest that our assumed cut point for defining “low femoral strength” is relevant clinically in terms of identifying those at a high risk of fracture. Further, using a much lower cut point of 2000 N to define low femoral strength also resulted in a higher prevalence than for the traditional clinical definition of osteoporosis using manufacturer young reference values for aBMD.

One notable finding was the greater percentage age-related reduction in the femoral strength of elderly women compared with the reduction in aBMD. Ideally, any such changes should be measured using a longitudinal study design because it is possible that historical influences of diet or activity level could produce an age dependence in a cross-sectional analysis that is not indicative of present-day rates of change.(32) However, the age- and sex-dependent estimated variations of femoral strength reported here from cross-sectional data are consistent with rates of change reported from 6-year longitudinal measurements of volumetric trabecular density at the lumbar spine, as measured by QCT for this cohort.(33) Further, the same BCT technique as used in this study was applied recently to a longitudinal observational study of ibandronate versus placebo (both groups receiving calcium and vitamin D) in osteoporotic postmenopausal women.(16) In that 12-month study, the average loss of femoral strength for the placebo group (data for 35 subjects, mean age approximately 64 years) was just under 4% (95% CI ∼1.5–6.2). The average value of femoral strength for that osteoporotic placebo group at baseline was about 2500 N. Such a value is typical of 78-year-old women in the community-sampled Rochester cohort, and our regression analysis indicated that the average annual percent loss of femoral strength for a 78-year-old was about 2.4%. This is statistically consistent with the finding from the ibandronate study. Although changes in femoral neck aBMD for the placebo group were not reported in that study, other much larger studies have reported annual reductions in total hip or femoral neck aBMD in placebo groups, in trials on postmenopausal women, of well under 1.0%.(34,35) Thus, biomechanically, there appear to be far greater annual reductions in femoral strength among those with already low femoral strength than previously suspected based on measured changes in DXA-derived aBMD.

The greater age-related decrease observed for femoral strength than for femoral neck aBMD indicates that the age dependence of aBMD underestimates the full effects of aging on femoral strength. There are a number of possible reasons why this is the case. First, in our computer models, based on observations from cadaver studies,(36) there is a nonlinear relation between changes in trabecular bone density and changes in trabecular bone strength such that changes in strength exceed changes in density. Second, as reported in an earlier cross-sectional analysis of this cohort,(18) changes in trabecular volumetric bone density over adulthood are almost twice as large as changes in cortical volumetric density. These differential changes in trabecular and cortical volumetric density together can result in changes in femoral neck aBMD that underestimate the true changes in volumetric density of the weakest bone within the femur, namely, the trabecular bone.

A third reason for the greater change in strength compared with aBMD is that DXA measures average bone density within a region of interest, including the thickness of the bone. By its 2D nature, DXA is relatively insensitive to focal bone loss, particularly in the femoral neck and intertrochanteric regions, where clinical fractures occur more frequently.(37) By contrast, such local weaknesses would substantially influence the BCT-derived measurement of bone strength because the finite-element model will fail in the locally weakest regions. As a result, the BCT approach can detect individuals at the very low end of the distribution of femoral strength who appear to have more normal values of femoral neck aBMD. This, and possible age-related differences in the relative loss of cortical and trabecular bone,(18,38) may explain why many osteopenic men in the MrOS fracture surveillance study who had a new hip fracture also had low femoral strength (<3000 N). Such averaging effects on bone density also have been noted in assessments of drug therapies. For example, in a recent BCT study of the biomechanical effects of parathyroid hormone after 1 year of treatment,(15) DXA-measured total hip aBMD did not change, whereas BCT-derived femoral strength increased. This occurred because trabecular density increased but cortical density decreased; the differential changes in the cortical and trabecular compartments had a canceling-out effect for total hip aBMD but a biomechanically net positive effect for femoral strength.

One other notable consequence of these findings is that sex-related differences in peak bone mass and in the subsequent annual rate of loss of femoral strength appear to be less important biomechanically than the earlier onset of strength loss that occurs among women. At age 85 years, we found that femoral strength, on average, was about 1000 N less for women than for men. The rate of loss of femoral strength, once it starts for both sexes, was about 6 N/year higher in women than in men; between the ages of 55 and 85 years, this corresponds to an accumulated strength deficit of 180 N. Thus, only about 18% of the sex-related strength deficit at age 85 resulted from sex-related differences in the rate of bone loss (once it starts). However, we also found that the annual loss of femoral strength in women (∼60 N/year) began about one decade earlier than in men. This decade-earlier onset of strength loss for women is important because it corresponds to an accumulated strength deficit of 600 N over the decade relative to men, which represents about 60% of the sex-related strength difference at age 85 years. The remaining 22% of this strength deficit at age 85 was due to differences in peak bone mass and any minor loss of strength before middle age. It follows, then, that women's bones are much weaker than men's bones in old age mainly because women begin to lose strength earlier—sex-related differences in peak bone mass and the rate of bone loss, though important, are much less so than this early-onset effect.

This study has a number of strengths and limitations. The primary strengths include the age-stratified nature of the study cohort, which was randomly sampled from the local population, and the use of the sophisticated BCT analysis technique, which provides a noninvasive estimate of femoral strength. While the data in Fig. 3 suggest that our cohort is representative of the larger US population, it is not clear how these trends extend to other populations in this country or elsewhere. An evaluation of nonwhite subjects using this approach would be of particular interest.(39) Moreover, our study is limited by the relatively small size of our cohort (∼50 subjects per sex per decade of age). It also remains to be seen from other fracture surveillance studies if our assumed prevalence threshold value of 3000 N for “low femoral strength” is as indicative of a high risk of hip fracture in women as it is in men. We note also that our measures of femoral strength are estimates and, though well validated in cadaver studies,(10,11) are based on models that do not include such patient-specific submillimeter characteristics as trabecular microarchitecture, collagen cross-linking, or alterations in the remodeling space.(8,40) While it is not clear if such characteristics play an appreciable role in clinical fracture risk assessment, the BCT measure of femoral strength used in this study has been shown to be highly predictive of incident hip fractures in elderly men.(17) Finally, although assessment of aBMD at the femoral neck is the preferred region for definition of osteoporosis,(41) we did not include total-hip, trochanteric, or spine aBMD measurements in this study when defining osteoporosis, nor did we address how inclusion of various other clinical factors such as age and history of previous fracture would alter the prevalence of those defined at high risk of hip fracture.(42) Including such parameters into the analysis represents an interesting follow-up study.

Our results may have clinical implications because they suggest that osteoporosis is underdiagnosed not only because an insufficient number of individuals are screened but also because DXA misses identifying many of those who are at high risk of fracture because of low femoral strength. Given the high prevalence of women with low femoral strength at advanced age, the paradigm of treating only those identified by DXA as being at high risk may be inherently flawed because it is more reactive than preventative. It may be that the optimal time to treat women should be sooner rather than later so as to prevent femoral strength from reaching such low levels in so many women. Since the rate of strength loss is relatively uniform with age once it starts, such early treatment might occur in the late 40s, 50s, or early 60s and potentially be equally effective for a given time course of treatment. Delaying the onset of bone loss in women should have even greater clinical impact because our calculations indicate that this early onset is the primary reason why women have such low bone strength compared with men at advanced age. While justifying any such strategies would require further study, the data presented here suggest that clinically relevant levels of low femoral strength are much more common in the general population than indicated by the prevalence of osteoporosis as currently defined by assessment of aBMD at the femoral neck.
